# Preparation of Soybean Fiber/Sodium Alginate Microgel and Its Application in Low-Fat Yogurt

**DOI:** 10.3390/foods13244156

**Published:** 2024-12-22

**Authors:** Cunshe Chen, Sihan Zheng, Zexun An, Zhihua Pang, Xinqi Liu

**Affiliations:** 1 Key Laboratory of Geriatric Nutrition and Health, Beijing Technology and Business University (BTBU), Beijing 100048, China; chencs@th.btbu.edu.cn (C.C.); zsh200103zsh@163.com (S.Z.); anzex09@163.com (Z.A.); liuxinqi@btbu.edu.cn (X.L.); 2 National Soybean Processing Industry Technology Innovation Center, Beijing Technology & Business University (BTBU), Beijing 100048, China

**Keywords:** soybean fiber, sodium alginate, microgel, oral lubrication

## Abstract

This study investigates the oral processing characteristics and application of soybean fiber and sodium alginate microgel in enhancing the texture and sensory attributes of low-fat yogurt. By combining soybean fiber with sodium alginate, a stable composite microgel system was developed with a uniform particle-size distribution. Oral lubrication performance was assessed by evaluating particle size, texture, friction coefficient and rheological properties, providing insights into how microgels improve food lubricity. The results showed that adding soybean fiber/sodium alginate microgel to low-fat yogurt significantly enhanced lubrication, texture and sensory quality compared to standard low-fat yogurt. The yogurt sample containing 2 wt% microgel achieved optimal sensory results, improving hardness and adhesiveness. This study suggests that soybean fiber/sodium alginate microgel offer a promising strategy for enhancing the sensory quality in low-fat dairy products, supporting healthier food innovations.

## 1. Introduction

Eating more high-fat foods is becoming more common as people’s material living levels rise [1,2]. The overconsumption of dietary fats is linked to various health problems, such as cardiovascular disease and obesity [3,4]. Therefore, the development of low-fat or skimmed food is a hot research field in the food industry. To satisfy market demand, a range of low-fat and non-fat products has appeared in the food industry. However, compared to full-fat foods, many low-fat or fat-free foods have poor performance in terms of taste, texture and appearance, resulting in lower consumer acceptance [5]. The use of cellulose as a fat substitute has been extensively studied in the development of many low-fat foods. Heggset et al. used enzyme-pretreated nanocellulose to substitute 9% of the fat in whole-fat mayonnaise without lowering the product’s viscoelasticity [6]. J. Velázquez et al. added ground-up cellulose nanofibers to ice cream, and this enhanced the low-fat samples’ sensory qualities [7]. Gibis et al. used microcrystalline cellulose to replace 50% of the fat in meat patties, which had a fat-like taste from the sensory point of view [8]. Rheology, texture and sensory qualities were the primary focus of the above studies on cellulose as a fat substitute; cellulose’s great mechanical strength and chemical inertness were the main constraints limiting its lubricity [9].

Oral tribology has been proved to be an important mechanism for the formation of oral sensory characteristics [10], which can be used to explain the behavior of food under conditions relevant to oral tribology and to describe the tactile sensation of food in the mouth [11]. By simulating the relative motion of tongue and palate, tongue and food in the oral cavity in vitro, the oral processing of food can be simulated. These factors also affect the size and trend of the friction coefficient of the sample [12]. Among them, the particle size affects the lubricity of fat substitutes in the food and beverage system. The particles will have a texture similar to fat when their diameter is less than 10 μm, which is below the minimum threshold for human oral mucosa perception of particles [13]. The size will affect the particle entrainment during the relative movement of the two contact surfaces. The particle entrainment depends on the size and characteristics of the gel particles [14,15], which can reduce or increase the friction coefficient between surfaces. Studies have shown that polymer particles trapped between two surfaces will produce special effects, thereby reducing the friction coefficient of the particle system. These phenomena have been analyzed and studied by tribology [16], and the relationship between the lubrication of lipid droplets and tribology has been established to a certain extent [11,17,18,19].

The sensory perception of fat in the human mouth is a complex process, involving the time-dependent deformation, structural damage and flow of food during chewing [20]. Fat-related sensory qualities, such as creaminess, smoothness, thickness and viscosity, are related to multiple parameters of a large number of rheological or texture experiments, but they are also difficult to quantify due to technical limitations [21,22]. During oral processing, fat components are deposited on the palate due to interaction with the oral epithelium [23]. At this time, the lubrication effect caused by fat in food can be evaluated by tribological tests [21,24,25]. Through the test of oral tribology, we can understand the information of oral sensory characteristics related to oily sensation and oral layer. Nguyen et al. conducted tribological measurements on cheeses with similar rheological properties but different fat content [20] and found that the extended regions of the boundary layer and the mixed layer of cheeses with different fat content were significantly different, and the samples with the highest fat content had the lowest friction coefficient. Therefore, tribology can be used to distinguish dairy products with similar rheological properties, and it can have a significant impact on the design of low-fat dairy products with similar lubrication behavior to full-fat emulsions.

The purpose of this study is to add sodium alginate to a soybean fiber gel system to prepare a stable and lubricating microgel, and to expand its application in improving the taste of low-fat food in combination with oral tribology to provide greater possibilities for industrial production.

## 2. Materials and Methods

### 2.1. Materials and Reagents

Soybean fiber was purchased from Shanghai Aladdin Biotechnology Co., Ltd (Shanghai, China). Food-grade sodium alginate and calcium carbonate were purchased from Tianjin Fu morning chemical reagent factory (Tianjin, China). Glucono-delta-lactone (GDL) was purchased from Anhui Xingzhou Medicine Food Co., Ltd (Anhui, China). Low-fat milk powder and whole milk powder were available from the local supermarket. Yogurt fermentation bacteria were purchased from Angel Yeast Co., Ltd (Hubei, China)., including *Lactobacillus delbrueckii subsp. bulgaricus* and *Streptococcus salivarius subsp. thermophilus*.

### 2.2. Methods

#### 2.2.1. Preparation of Soybean Fiber Suspension

The soybean fiber, water and zirconia beads were placed in a planetary ball mill of JX-4G (Jingxin Instruments, Shanghai, China) according to a certain proportion [26,27]. Two kinds of zirconia beads with diameters of 3.2 mm and 5.2 mm were selected, and the number ratio was 5:1. An appropriate amount of soybean fiber powder and zirconia beads were weighed at a mass ratio of 1:22, and deionized water was added to make the fiber concentration 15.63 wt%. Then the soybean fiber powder and zirconia beads were ball milled using a planetary ball mill. The rotation speed of planetary ball milling was set to 670 rpm, and the work was stopped for 30 min every 2 h, and the total working time was 12 h.

The prepared soybean fiber homogenate was diluted to 1.0 wt%, homogenized five times with a high-pressure homogenizer at 5000 psi, and then the solution was centrifuged at 2000 rpm for 10 min using a X-30 benchtop centrifuge (Beckman Coulter, California, USA). The supernatant was discarded, and the precipitate was collected. The N-1200B rotary evaporator (EYELA, Tokyo, Japan) was run for one hour at a temperature of 35–40 °C and a rotational speed of 80 rpm.

To get rid of the fiber remnant from the rotary evaporation, enough samples of soybean fiber with a higher concentration were regularly collected and sieved through an 80-mesh sieve. Following an appropriate rotary evaporation, the samples were averagely split into three sections, and each section’s initial mass was noted. After that, the samples were dried in an oven set to 60 °C until the mass no longer decreased. The precise value was then read out, and the final soybean fiber concentration was determined. A known quantity of soybean fiber was diluted with deionized water and uniformly swirled to create four distinct soybean fiber suspension concentrations: 5 wt%, 10 wt%, 20 wt% and 40 wt%.

#### 2.2.2. Preparation of Soybean Fiber/Sodium Alginate Microgel

Sodium alginate at concentrations of 1.5 wt%, 2.1 wt%, 2.7 wt% and 3.0 wt% was heated from 80 °C to 90 °C and uniformly agitated to completely dissolve the alginate and create a viscous solution. The sodium alginate solution was cooled and stored at 4 °C. The soybean fiber was combined with four different concentrations of sodium alginate solution, which were 0.5 wt%, 0.7 wt%, 0.9 wt% and 1.0 wt%, respectively. The 15:30 mM calcium carbonate and GDL were added and stirred evenly. After standing overnight, a stable gel system was formed.

#### 2.2.3. Preparation of Yogurt

Separately, 40 g of whole milk powder and 40 g of low-fat milk powder was added to 100 mL of pure water, heated and dissolved to obtain whole milk and low-fat milk. White granulated sugar (7.0 wt%) was then added.

After cooling to room temperature, soybean fiber/sodium alginate microgel concentrations of 1 wt% and 2 wt% were added to low-fat milk; 3.0 wt% yogurt fermentation powder was added to the fermentation process at 42 °C. Following uniform mixing, ten hours of heating time were maintained. After fermentation, the yogurt was placed in a 4 °C refrigerator. The low-fat yogurts with 1 wt% and 2 wt% microgel concentrations were studied. Whole milk yogurt without microgel and low-fat yogurt without microgel were studied as control groups.

#### 2.2.4. Particle-Size Analysis

The particle-size distribution was assessed utilizing a laser particle-size analyzer SALD-2300 (Shimadzu Corporation, Tokyo, Japan) [28]. The refractive indices for the sample and water were set as 1.45 and 1.33, respectively. The results were presented as particle-size distribution and average particle size. The samples were dispersed in distilled water by vortex oscillation. The sample was circulated in the sampling unit with water and measured at ambient temperature.

#### 2.2.5. Rheology

Sample viscosities were measured using a DHR-1 rheometer (TA Instruments, Elstree, USA) under the following test conditions: temperature set at 37 °C, stainless steel cone plate geometry (diameter: 40 mm, cone angle: 2°) with a 57 μm gap and a shear rate ranging from 0 to 100 s^−1^ [29]. The viscosity change with the shear rate was recorded. An appropriate amount of sample was added to the sample table, the Gap value set to 59 μm, trimmed, and the Gap value adjusted to 57 μm and then measured. Each sample was subjected to three independent repeated analyses, and the results were averaged and standard deviation established.

#### 2.2.6. Oral Tribology

The lubricating properties of the samples were measured using a TA rheometer equipped with three balls on plate tribo-rheometry (TA Instruments, Elstree, USA). Polydimethylsiloxane (PDMS) with a surface roughness (Ra) of less than 50 nm and a contact angle of 95.33° was prepared according to the product specifications and used as the lower contact surface to simulate the tongue surface [29]. The PDMS and crosslinker were mixed in a 10:1 ratio, vacuumed to remove trapped air and cured at 90 °C for 22 h. The PDMS and crosslinker were used to simulate the lower contact surface of the tongue. Before each test, the surface was cleaned with ultrapure water. Measurements were conducted at 37 °C with a constant normal force of 1 N. The coefficient of friction was recorded at entrainment speeds ranging from 0.1 to 300 μm/s. For each test, 4 g of the sample was gently spread to cover the base, and the coefficient of friction was measured across the specified range of entrainment velocities. Three independent replicates were performed for each sample, and the results were averaged and standardized.

#### 2.2.7. Texture

The sample container was put immediately on the CT3 texture analyzer (Brookfield, Middleboro, MA, USA) bottom plate for determination after being left at 4 ° C for the entire night. A cylindrical 12.7 mm diameter probe was employed. The temperature was 4 °C; the test distance was 10 mm; the trigger force was 5 g; and the test speed was 0.5 mm/s constant speed operation. The stickiness and first cycle hardness were noted.

### 2.3. Sensory Evaluation

#### 2.3.1. Selection of Panelists

This study organized a team of 8 professionally trained panelists, including 4 males and 4 females, all of whom had relevant food professional backgrounds. The panelists had to pass tests, including basic sensory recognition ability, sensory sensitivity and description ability tests. All panelists were in good health, with no smoking habits and not in pregnancy or lactation.

#### 2.3.2. Selection of Terms

Attributes were selected and discussed in an open session with the panel leader, and a unique list of attributes was established by consensus (Table 1). The assessors were first given a brief outline of the procedures, a list of attributes and representative samples. They were then asked to choose and write down the most appropriate attributes to describe all the sensory properties of the yogurt or to suggest new ones. The panel leader collected all the attributes and wrote them on a board, and the panel discussed the appropriateness of the selected attributes, their definitions and preliminary methods for assessing the products. By the end of this session, a consensus on the list of attributes was reached: color, flavor, mouthfeel, texture and taste.

#### 2.3.3. Panel Training

The training involved two days, during which each attribute was presented to the panelists along with definitions and physical references to facilitate their understanding. Panelists had to learn how to accurately identify and record sensory attributes to ensure the unity of sensory states before testing. The panelists had sufficient relevant knowledge and sensory evaluation ability to evaluate the samples objectively and accurately.

In addition, all panelists were required to drink only pure water within 2 h before the experiment, and any other food or beverage was prohibited. The sensory evaluation experiment was carried out in the specialized laboratory of Beijing Technology and Business University.

#### 2.3.4. Formal Assessment

During the data collection part, the samples were presented one by one, in blind, in a completely balanced order, each on a separate plastic tray labeled with a random three-digit code. All participants were required to read and sign an informed consent form before the start of the test. Their participation in the study and personal data in the study were confidential. Before obtaining the participants’ permission, it was agreed that any information that identified the participants would not be disclosed to members outside the research team. All research members and research stakeholders will keep all participants’ identity confidential as required. When the results of this study are published, no information about their identity will be disclosed. Even if a participant agrees to participate, they can change their mind at any time and tell the researcher to withdraw from the study.

Panelists were instructed to rinse their mouths with water between each sample evaluation to minimize interference, with 1 min time breaks in between sample. The evaluation of each sample was repeated three times to ensure the reliability and repeatability of the data. Moreover, the three replicates were performed on three different days.

### 2.4. Statistical Analysis

All measurements were conducted on three independent replicates, and the results were presented as mean and standard deviation. Statistical analysis software (SPSS 25.0, Chicago, IL, USA) was used to analyze variance (ANOVA), with a pairwise comparison of means using the Tukey HSD post hoc test. The significance of the results was determined at a significance level of *p* < 0.05. A graphical software program (Origin 8.5.1, origin, Northampton, MA, USA) was used to draw the graphs.

## 3. Results and Discussion

### 3.1. Effects of Different Concentrations of Soybean Fiber and Soybean Fiber/Sodium Alginate Microgel on Oral Friction

#### 3.1.1. Tribological Properties

**Figure 1:**
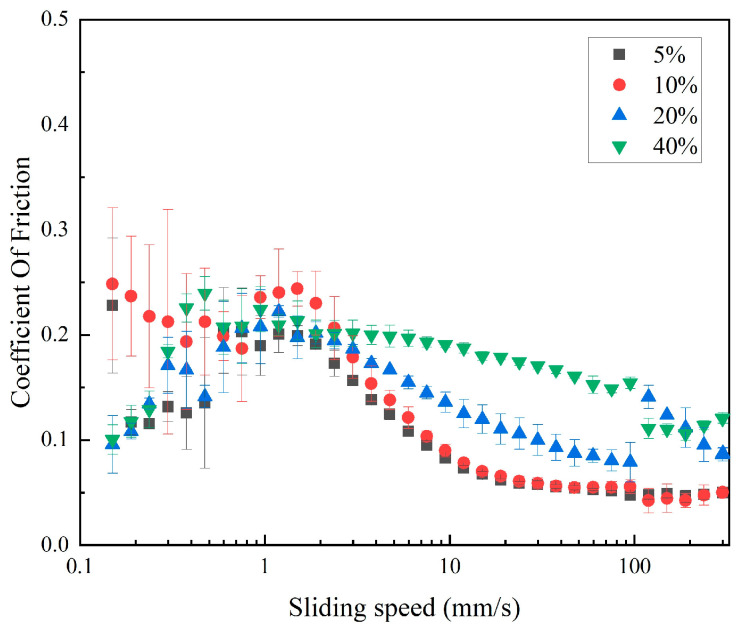
Coefficient of friction of soybean fiber with different concentrations.

The friction coefficient curves of the soybean fiber suspension with the change in sliding speed were shown in Figure 1. When the percentage of soybean fiber increases between 5 and 40 percent, the lubricity falls, and the friction coefficient rises.

This may be due to the high concentration of soybean fiber [30], and the aggregation state is serious and may have an adverse effect on the contact area [31]. Ago et al. also found that ball milling with water can transform the original fibrous fibers into aggregated spherical particles when studying the effect of different solvents on the morphology of cellulose under ball milling conditions [32]. The soybean fiber suspension’s total friction coefficient ranged from 0.0 to 0.3, with a lower coefficient and superior lubricating effect at sliding speeds of 0.1 to 1 mm/s. The curve exhibited a declining tendency before eventually tending to flatten out when a sliding speed of 2 mm/s was used as the inflection point. Except for 10 wt%, the friction coefficient of other concentration samples increased first and then decreased in a small range. The concentration of soybean fiber has an effect on the friction coefficient, and the selection of appropriate concentration is very important to optimize the lubrication performance of the product. In the low-speed sliding range, the state of the 5 wt% and 10 wt% concentration samples was unstable, so the soybean fiber concentration used in the subsequent experiments was 20 wt%. It may be that the cellulose at this concentration exposes more hydroxyl groups after microfluidic treatment, has greater electrostatic repulsion, and the suspension is more stable [33].

**Figure 2:**
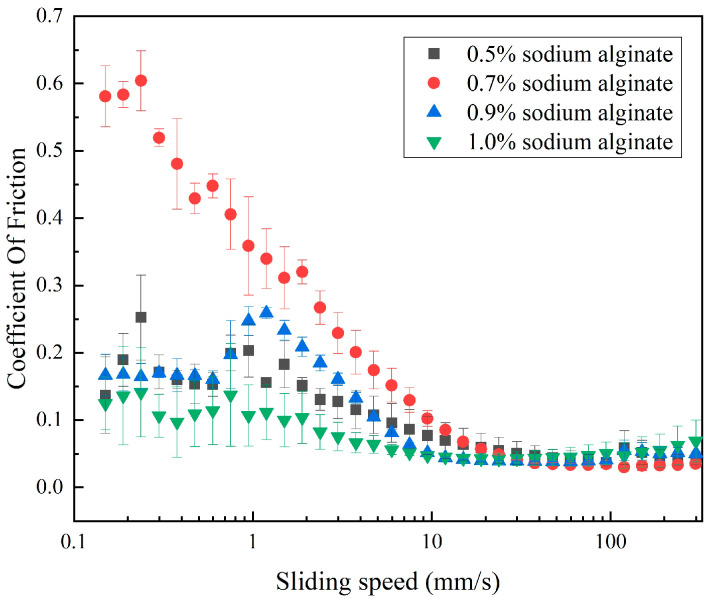
Coefficient of friction of microgel with different sodium alginate concentrations.

The variation curves of the friction coefficient of microgel suspensions with different gel strengths with sliding speed are shown in Figure 2. A border lubrication zone is seen in all microgels except the 0.7 wt% sample when the sliding speed is between 0.1 and 1 mm/s. Although the sliding speed is now low, and the number of particles entering the friction is insufficient to separate the contact surface, certain variances do exist. Aggregation of the microgel particles is caused by the lowness of the hydrodynamic forces in the space between their contact surfaces [34]. The sample will progressively enter the interface to create a layer of lubricating film as the sliding speed increases, and the friction coefficient reaches its maximum at 2 mm/s. With an increasing sliding speed, the friction coefficient starts to drop and moves into the hydrodynamic zone, where the solution’s friction effect is mostly influenced by the sample’s viscosity. It then tends to level out.

The line of the friction coefficient of microgel at a 1 wt% concentration was more stable than that of the other three. This may mean that a 1 wt% concentration of microgel is more effective in forming a lubricating film and provides a better lubrication performance. This difference may be related to the gel strength of the microgel and the interaction between particles. Microgel with a 1 wt% sodium alginate concentration may have a better particle distribution and network structure [30], thus providing a more stable lubrication effect during friction.

#### 3.1.2. Particle-Size Distribution

**Figure 3:**
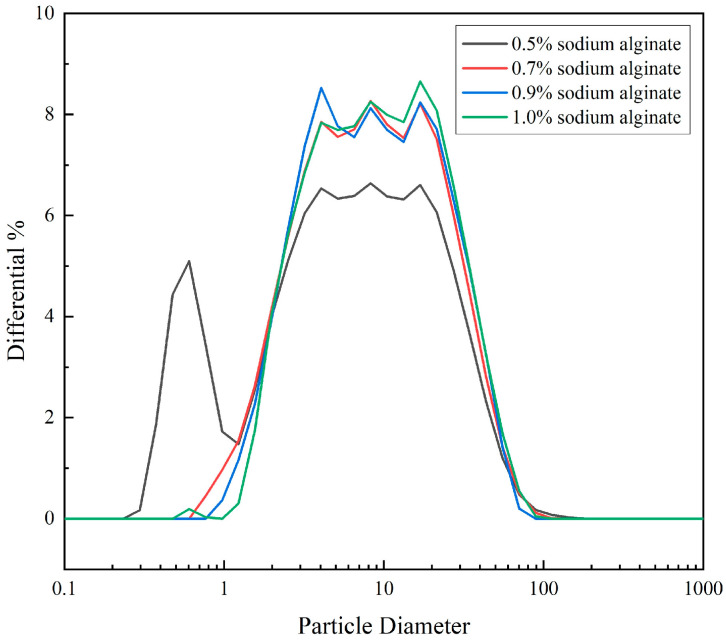
Particle-size distribution of microgel with different sodium alginate concentrations.

It is generally believed that particle-size distribution is an indicator of colloidal stability [35]. According to the results shown in Figure 3, by studying the particle-size distribution of microgel with different concentrations, the average particle size of soybean fiber microgel with a sodium alginate concentration of 0.5 wt% was 5.149 ± 0.46 μm, and the average particle size was small, but the particle-size distribution range was wide and uneven. With a concentration of 0.7 wt%, the average size of the microgel particles was 6.689 ± 0.61 μm; with a concentration of 0.9 wt%, the average size was 6.888 ± 0.70 μm; and with a concentration of 1.0 wt%, the average size was 6.750 ± 1.20 μm. At this concentration, the average particle size of the microgel was small, and the distribution was relatively average.

A particle-size analysis is often used to indicate colloidal stability, with smaller sizes indicating better stability [36]. The particle size affects the appearance, flavor and taste of the dairy product. The roughness and graininess of the dairy product increase with a growing particle size, whereas the stability of the product is improved by decreasing the particle size. Previous studies have also found that small particles exhibit a better lubrication performance than large particles [37]. Combined with the previous tribological results, the soybean fiber/sodium alginate microgel with 1 wt% sodium alginate content was selected for subsequent research. This choice was not only based on the uniformity of the particle-size distribution, but also took into account the combined effect on the taste and stability of the product. By adjusting the concentration of sodium alginate, the performance of soybean fiber/sodium alginate microgel can be optimized to meet specific food application needs.

### 3.2. Application of Microgel in Low-Fat Yogurt

#### 3.2.1. Tribology of Soybean Fiber/Sodium Alginate Microgel in Low-Fat Yogurt

**Figure 4:**
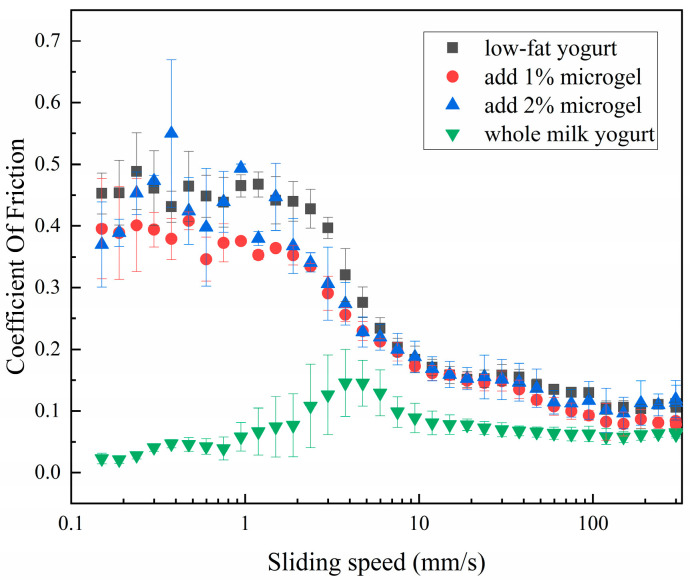
Coefficient of friction of low-fat yogurt with different microgel concentrations.

Oral tribology studies the friction behavior and lubrication characteristics between the interacting surfaces in the oral environment, which provides important information for understanding the interaction between food and oral surface [8,9,10]. Therefore, it has a certain application value for the evaluation of yogurt. We studied the effect of adding soybean fiber/sodium alginate microgel to different content levels on the oral friction coefficient and lubrication performance of low-fat yogurt. Figure 4 shows the change curve of the friction coefficient of the whole milk yogurt and the low-fat yogurt samples with different microgel content. We found that the friction coefficient of low-fat yogurt without soybean fiber/sodium alginate microgel was the largest. When it was between 0.1 and 5 mm/s, the friction coefficient of the two groups of samples with different concentrations of soybean fiber/sodium alginate microgel was smaller than that of low-fat yogurt but larger than that of whole milk yogurt. This improvement may be attributed to the lubrication film formed by the microgel in the mouth, which reduces the friction between the microgel and the surface of the mouth [38], thus providing a taste closer to whole milk yogurt, which can mainly be attributed to a “ball bearing” effect [39]. However, an excess of microgels could lead to their jamming and compression, which inhibits the rolling motion of the microgels, thereby increasing the friction. Similar observations have been reported for gelatin microgels [40] and whey protein–xanthan gum microgels [41]. This part of the results proved that soybean fiber/sodium alginate microgel had a positive effect on the oral lubrication performance of low-fat yogurt.

#### 3.2.2. Rheology of Soybean Fiber/Sodium Alginate Microgel in Low-Fat Yogurt

**Figure 5:**
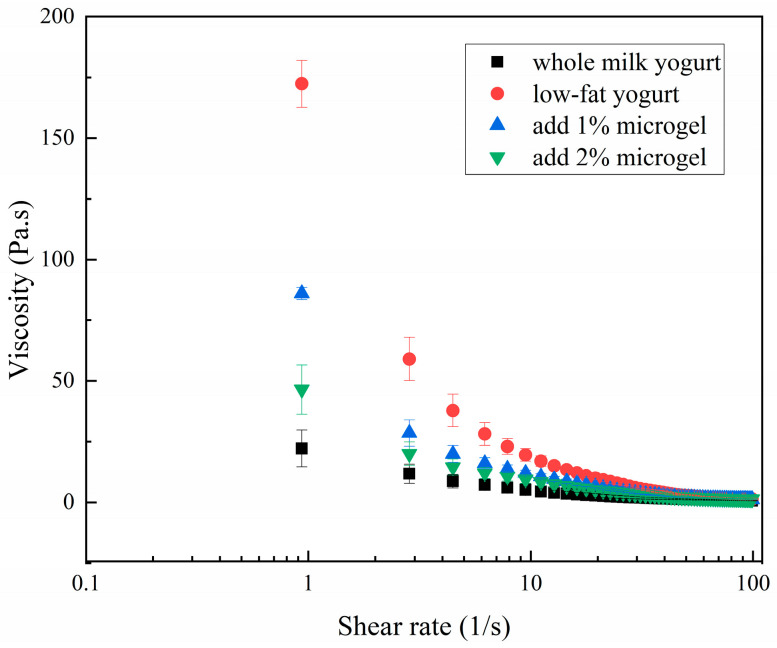
Viscosity of low-fat yogurt with different microgel concentrations.

Figure 5 shows the change in viscosity of yogurt with shear rate at different concentrations. All four samples showed shear thinning behavior, that is, with the increase in shear rate, the apparent viscosity decreased. The viscosity of low-fat yogurt was lower than that of the other three samples. The viscosity of the two groups of low-fat yogurt with soybean fiber/sodium alginate microgel added was higher than that of the control group, which was due to the fact that the entangled fiber network had a certain anti-flowability [42]. As the shear rate increases, the entangled network structure is broken, the fibers are separated from each other, and the arrangement gradually becomes more regular, which promotes the flow of the sample and leads to a decrease in viscosity. As the concentration of soybean fiber/sodium alginate microgel increases, a higher level of interaction is formed in the solution environment with more fiber numbers, increasing fluid resistance and viscosity [43].

#### 3.2.3. Texture of Soybean Fiber/Sodium Alginate Microgel in Low-Fat Yogurt

**Figure 6:**
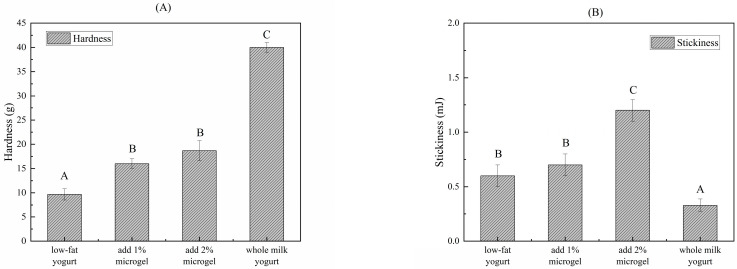
Texture of low-fat yogurt with different microgel concentrations. (**A**) Hardness and (**B**) stickiness. Bars within one attribute with different letters denote a statistically significant difference (*p* < 0.05).

Figure 6 displays the impact of soybean fiber/sodium alginate microgel on the textural characteristics of low-fat yogurt, including important markers of hardness and stickiness. The texture results clearly show that adding soybean fiber/sodium alginate microgel to low-fat yogurt has increased its hardness (Table 2). The microgels have increased the hardness of low-fat yogurt beyond that of low-fat blank yogurt. Low-fat yogurt’s hardness can be considerably increased by adding soybean fiber/sodium alginate microgel, even at low concentrations. At 2 wt%, the hardness is nearly as high as that of whole milk yogurt. This demonstrates how adding microgel can mimic the function of fat in yogurt and improve the product’s stability and flavor. The stickiness of low-fat yogurt with soybean fiber microgel added was higher than that of low-fat yogurt and whole milk yogurt. This shows that soybean fiber/sodium alginate microgel can effectively improve the stickiness of low-fat yogurt, and the heat treatment temperature of fermented yogurt is 42 °C, which may help the microgel to play a better role. This thickening effect is beneficial in improving the taste and stability of yogurt, especially in dairy products [44]. Stickiness is one of the important indicators for consumers to experience the taste of products. 

In addition, this improved texture property may also have a positive impact on the processing and storage of the product, as better texture characteristics may help reduce breakage and separation during transport and storage [45]. The above results show that the addition of soybean fiber/sodium alginate microgel helps improve the texture of the low-fat yogurt, probably by making the yogurt network structure more resilient [45,46], making it more delicate and smoother in the oral processing stage. This is a positive improvement for consumers pursuing a healthy diet, as it provides an eating experience closer to whole milk yogurt, while reducing fat content compared to whole milk yogurt.

#### 3.2.4. Sensory Evaluation of Soybean Fiber/Sodium Alginate Microgel Low-Fat Yogurt

**Figure 7:**
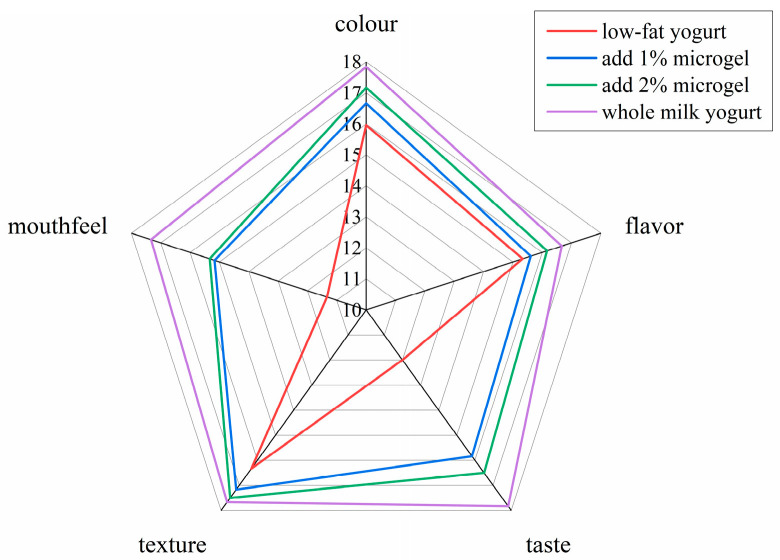
Sensory evaluation of low-fat yogurt with different microgel concentrations.

To create a sensory evaluation group, eight individuals with professional backgrounds in food and experience in sensory evaluation were invited and assessed. After a while, a tiny quantity of liquid supernatant was noticed in each of the four sets of yogurt samples. The samples were homogeneous and without any odd smells, stratification or precipitation phenomena. The results of the sensory evaluation of all samples were as shown in Figure 7. The scores of each item of the two groups of samples with different concentrations of microgel were lower than those of whole milk yoghurt but better than those of the low-fat yogurt control group, and the color and texture were similar. The best outcomes from the four sample groups were the lubrication and taste of whole milk yogurt when it enters the human mouth. The findings demonstrated that the addition of soybean fiber/sodium alginate microgel enhanced the low-fat yogurt’s flavor, taste and food acceptability while having no influence on color or texture. This may be due to the fact that microgel enhances the viscosity and viscoelasticity of yogurt, which allows sodium alginate to be utilized as a bio-lubricant to improve the taste of food [47].

Figure 7 presents the sensory evaluation results of low-fat yogurt with different microgel concentrations. The data in this figure highlight the potential of soybean fiber/sodium alginate microgel in improving the sensory quality of low-fat yogurt, providing a scientific basis for the sensory enhancement of low-fat dairy products. By adjusting the amount of the microgel concentration, the sensory characteristics of low-fat yogurt can be optimized to better meet consumer demands for both health benefits and taste.

## 4. Conclusions

This study investigated the application of soybean fiber/sodium alginate microgel in low-fat yogurt to enhance oral lubrication, texture and sensory quality. By combining soybean fiber with sodium alginate, a composite microgel was developed with a relatively uniform particle size, offering favorable properties for food applications. Evaluations of particle size, rheology and friction characteristics revealed that these microgels significantly improved the physical properties of low-fat yogurt, mimicking the desirable mouthfeel and texture of full-fat dairy products.

Soybean fiber/sodium alginate microgels, particularly at a 2 wt% concentration, were found to enhance yogurt’s mouthfeel by reducing friction and improving creaminess and smoothness. This concentration provided an optimal balance of sensory qualities, including increased hardness, adhesiveness and mouth-coating properties that contributed to a stable structure and a creamier, richer experience. Additionally, the microgel exhibited shear-thinning behavior, supporting a better flow and spreadability during oral processing, which further enhanced the perception of creaminess and lubrication. Overall, this study highlights the promise of soybean fiber/sodium alginate microgel for developing reduced-fat foods that maintain high sensory standards, providing a new approach for healthier, consumer-preferred dairy alternatives. Future work could explore their application across various food systems to broaden their impact in low-fat food innovation.

## Figures and Tables

**Table 1:** Standards of sensory evaluation of yogurt.

Items	Evaluation Standard	Score
Color	The color is uniform, milky white	15–20
	The color is basically uniform, milky white color	10–15
	Local uneven color, milky white color	5–10
	The overall color is not uniform, the color is not normal	<5
Flavor	Rich and harmonious aroma, no peculiar smell	15–20
	The fragrance is rich, no peculiar smell	10–15
	The fragrance is weak, no peculiar smell	5–10
	The fragrance is weak, peculiar smell	<5
Texture	Elastic texture, trace or no supernatant precipitation, no bubbles, stratification or precipitation phenomenon	15–20
	The texture is elastic, a small amount of supernatant is precipitated, and a small amount of bubbles, stratification or precipitation appear	10–15
	The elasticity is poor, the supernatant precipitates more, and there are obvious bubbles, stratification or precipitation phenomena	5–10
	Turbid soft, flocculent or cavitation phenomena, stratification or precipitation is obvious	<5
Taste	The sour taste is moderate, no astringent, bitter, salty and other odors	15–20
	Taste slightly sour, no astringent, bitter, salty and other smells	10–15
	The sour taste is obvious, no astringent, bitter, salty and other odors	5–10
	The sour taste is obvious, with astringent, bitter, salty and other odors	<5
Mouthfeel	The taste is delicate and smooth, no graininess	15–20
	The taste is delicate, slightly grainy	10–15
	The taste is slightly rough, with graininess	5–10
	The taste is rough, with obvious graininess	<5

**Table 2:** The apparent viscosity is reported at a fixed shear rate of 50 s^−1^. Textural parameters of yogurt containing different levels of microgels.

Samples	Apparent Viscosity (Ps s)	Hardness (g)	Stickiness (mJ)
Whole milk yogurt	1.33 ± 0.24 ^A^	40.00 ± 1.00 ^C^	0.33 ± 0.06 ^A^
Low-fat yogurt	2.85 ± 0.21 ^C^	9.67 ± 1.15 ^A^	0.60 ± 0.10 ^B^
Add 1% microgel	2.04 ± 0.01 ^B^	16.00 ± 1.00 ^B^	0.70 ± 0.10 ^B^
Add 2% microgel	2.12 ± 0.29 ^B^	18.667 ± 2.08 ^B^	1.20 ± 0.10 ^C^

^A–C^ Mean values with different letters within columns are significantly different (*p* < 0.05).

## Data Availability

The original contributions presented in the study are included in the article, further inquiries can be directed to the corresponding author.
